# Community knowledge, attitudes and practices (KAP) on malaria in Swaziland: A country earmarked for malaria elimination

**DOI:** 10.1186/1475-2875-8-29

**Published:** 2009-02-19

**Authors:** Khumbulani W Hlongwana, Musawenkosi LH Mabaso, Simon Kunene, Dayanandan Govender, Rajendra Maharaj

**Affiliations:** 1 Malaria Research Lead Programme, South African Medical Research Council, 491 Ridge Road, Overport, Durban, 4001, South Africa; 2 Malaria Control Programme, Department of Health, Nkoseluhlaza Street, PO Box 53, Manzini, Swaziland

## Abstract

**Background:**

The potential contribution of knowledge, attitudes and practices (KAP) studies to malaria research and control has not received much attention in most southern African countries. This study investigated the local communities' understanding of malaria transmission, recognition of signs and symptoms, perceptions of cause, treatment-seeking patterns, preventive measures and practices in order to inform the country's proposed malaria elimination programme in Swaziland.

**Methods:**

A descriptive cross-sectional survey was undertaken in four Lubombo Spatial Development Initiative (LSDI) sentinel sites in Swaziland. These sentinel sites share borders with Mozambique. A structured questionnaire was administered to 320 randomly selected households. Only one adult person was interviewed per household. The interviewees were the heads of households and in the absence of the heads of households responsible adults above 18 years were interviewed.

**Results:**

A substantial number of research participants showed reasonable knowledge of malaria, including correct association between malaria and mosquito bites, its potential fatal consequences and correct treatment practices. Almost 90% (n = 320) of the respondents stated that they would seek treatment within 24 hours of onset of malaria symptoms, with health facilities as their first treatment option. Most people (78%) perceived clinics and vector control practices as central to treating and preventing malaria disease. Indoor residual spraying (IRS) coverage and bed net ownership were 87.2% and 38.8%, respectively. IRS coverage was in agreement with the World Health Organization's (WHO) recommendation of more than 80% within the targeted communities.

**Conclusion:**

Despite fair knowledge of malaria in Swaziland, there is a need for improving the availability of information through the preferred community channels, such as *tinkhundlas *(districts), as well as professional health routes. This recommendation emerges along with the documented evidence suggesting that as the level transmission and disease decreases so does the perception about the importance of malaria control activities. Finally, given the relatively moderate ownership of bed net there is a need for future studies to evaluate the distribution of insecticide-treated nets (ITNs) compared with IRS.

## Background

Malaria remains a major cause of morbidity and mortality in tropical and subtropical regions of the world, despite decades of malaria control efforts. There are approximately 300–500 million clinical cases and about one million deaths due to malaria globally, and Africa south of the Sahara accounts for over 90% of the disease burden [1]. Except for southern Africa, many countries in the continent do not have successful malaria control programmes due to among others the magnitude of the problem compounded by lack of adequate health infrastructure, as well as financial and human resources [2]. Nevertheless, hope has been rekindled by the renewed interest in scaling up the implementation of proven interventions across the continent [3], and even elimination is being targeted where malaria has been reduced to very low levels [4].

In most southern African countries, malaria has been greatly reduced through the use of indoor residual spraying complemented by effective case management [2]. In the south-eastern region, recent achievements are largely attributed to the successes of the Lubombo Spatial Development Initiative (LSDI), a collaborative approach to malaria control between Mozambique, Swaziland and South Africa [5]. Consequently, the Southern African Development Community (SADC) is one of the few regions in Africa with the potential to eliminate malaria [6]. Swaziland is among the countries earmarked as ready for malaria elimination phase in the SADC region. Elimination requires great focus on malaria transmission foci at a local level and community understanding of malaria can greatly improve the realisation and sustainability of this strategy. However, in more than 50 years of malaria control in Swaziland no published survey of the community knowledge, attitudes and practices has been conducted.

This study investigates the local communities' understanding of malaria transmission, recognition of signs and symptoms, perceptions of cause, treatment-seeking patterns, preventive measures and practices in Swaziland in order to inform the country's proposed malaria elimination programme.

## Methods

### Study area

Swaziland is a landlocked country located in south-eastern Africa, between Mozambique and South Africa with an estimated population of 1.2 million. About one-third (400,000) of the Swaziland population are at risk of malaria infection [6]. Malaria transmission is seasonal and unstable with the peak between April and May. Malaria is a notifiable medical condition and cases are passively and actively detected. Passively detected cases are those that present themselves to clinics or hospitals with signs and symptoms of malaria. Actively-detected cases are people that have a recent history of illness indicative of malaria but did not go to any health facility and are traced and detected by surveillance agents. IRS with insecticides, mainly dichlorodiphenyltrichloroethane (DDT) is the mainstay of malaria control supplemented by effective case management and insecticide-treated nets (ITNs) [6].

**Figure 1:**
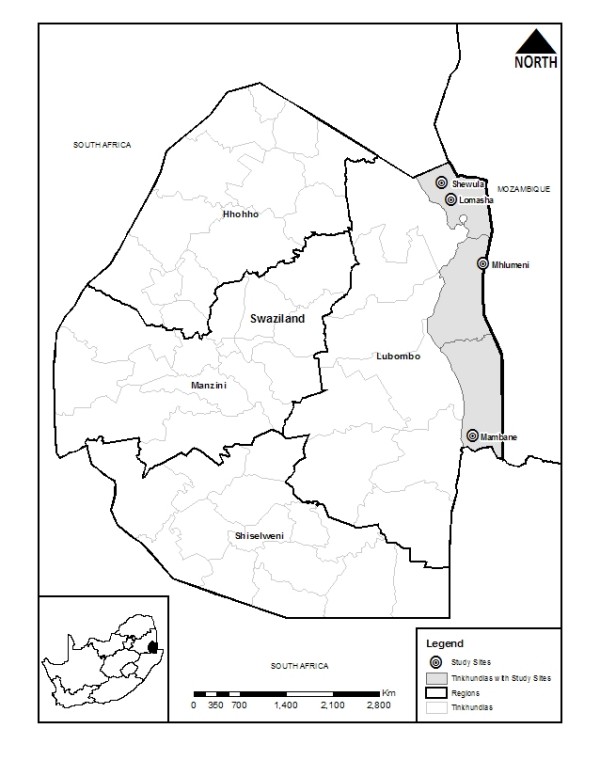
**Map of Swaziland showing Tinkundlas (districts) where study sites are located and the insert shows the position of the country within the Lubombo region**.

Study sites selected for this survey are situated in the Lubombo region within the three *tinkhundlas *(districts), Lomahasha, Siteki and Matsanjeni-Mambane sharing borders with Mozambique. The sites include Lomahasha, Mambane, Mhlumeni and Shewula (Figure 1). These sites are part of the Lubombo Spatial Development project and are located in former high risk area where transmission has been greatly reduced due to the interventions of this project [5]. In Swaziland, treatment is given on a presumptive basis at a clinic level due to infrastructural constraints. However, laboratory confirmation is required at a health centre (health facility above clinic but below hospital capacity) and hospital levels. Chloroquine remains the first-line drug for uncomplicated malaria as well as the prevention of malaria in pregnancy. Sulphadoxine-pyrimethamine is a second-line drug in cases of first-line drug treatment failure and the quinine is recommended for severe malaria.

### Study design and data collection

The study was a descriptive cross-sectional survey. A structured questionnaire was designed and administered by trained field workers. The first part of the questionnaire included demographic characteristics, whereas the second part had questions on, adult residents' attitudes and understanding of malaria transmission, recognition of signs and symptoms, perceptions of cause, treatment-seeking patterns, preventive measures and practices. The questionnaire was translated into SiSwati and piloted before being administered (Additional File 1 shows questionnaire in English).

The questionnaire was administered to 320 randomly selected households for two weeks in July 2007. The head of household or a responsible adult was interviewed. Only one person per household was interviewed. All participants gave verbal consent. Questionnaire administration was monitored daily for quality control. Data collected were double entered into Microsoft Access database and descriptively analysed using Epi-Info version 3.3.2.

## Results

The study participants consisted of 48.4% males and 51.6% females. Demographic data revealed that the ages for the households' members in the selected areas ranged from less than one year to 99 years with the mean, median and standard deviation of 23.3, 19 and 18.8, respectively. However, only adults above the age of 18 years were interviewed. Table 1 shows the demographic characteristics of the study population including the level of education, occupation and households' sizes. The majority of the participants had only acquired primary education and few had tertiary qualifications. When excluding students (29.3%), the vast majority of people were unemployed. On average most households had six members, and ranged between one and 19 people per household. In total 5.8% (n = 1971) people had suffered from malaria during the period of January to mid July 2007.

**Table 1:** Demographic characteristics of persons in the selected study households in the Lubombo region, Swaziland, mid 2007.

***Characteristics***	***n***	***%***
**Gender**		
Male	954	48.4
Female	1017	51.6
**Age groups in years**		
0 – 5	323	16.4
6 – 15	496	25.2
16 – 40	784	39.8
41+	341	17.3
Not known	27	1.4
**Number of persons in household**		
1 – 4	105	32.8
5 – 9	169	52.8
10 or more	46	14.4
**Highest level of education completed**		
No education	327	16.6
Primary	762	38.7
Secondary	489	24.8
Tertiary Qualification	25	1.3
Below school-going age	310	15.7
Don't know	16	0.8
Other	42	2.1
**Occupation of each person**		
Minor	353	17.9
Housewife	198	10.0
Pensioner	37	1.9
Other	98	5.0
Unemployed	473	24.0
Farm worker	57	2.9
Trained employee	40	2.0
Self Employed	88	4.5
Civil servant	46	2.3
Studying	578	29.3
Don't know	3	0.2

In terms of household structure (Table 2) cement blocks followed by stick and mud were the most prevalent types of house walls in the surveyed localities. There was also a considerable number of structures with stones and cement as well as stones and mud walls. Most roof types were zinc and grass.

**Table 2:** Types of household structures among the selected study households in the Lubombo region, Swaziland.

***Household structure***	***n***	***%***
**Type of wall**		
Cement blocks	181	56.6
Clay or clay blocks	9	2.8
Fire bricks	4	1.3
Stones and cement	85	26.6
Stones and mud	93	29.1
Stick and mud	164	51.3
**Type of roof**		
Grass	237	74.1
Tiles	4	1.3
Zinc	245	76.6
Canvas	3	0.9

### Malaria knowledge – Information Education and Communication (IEC)

**Figure 2:**
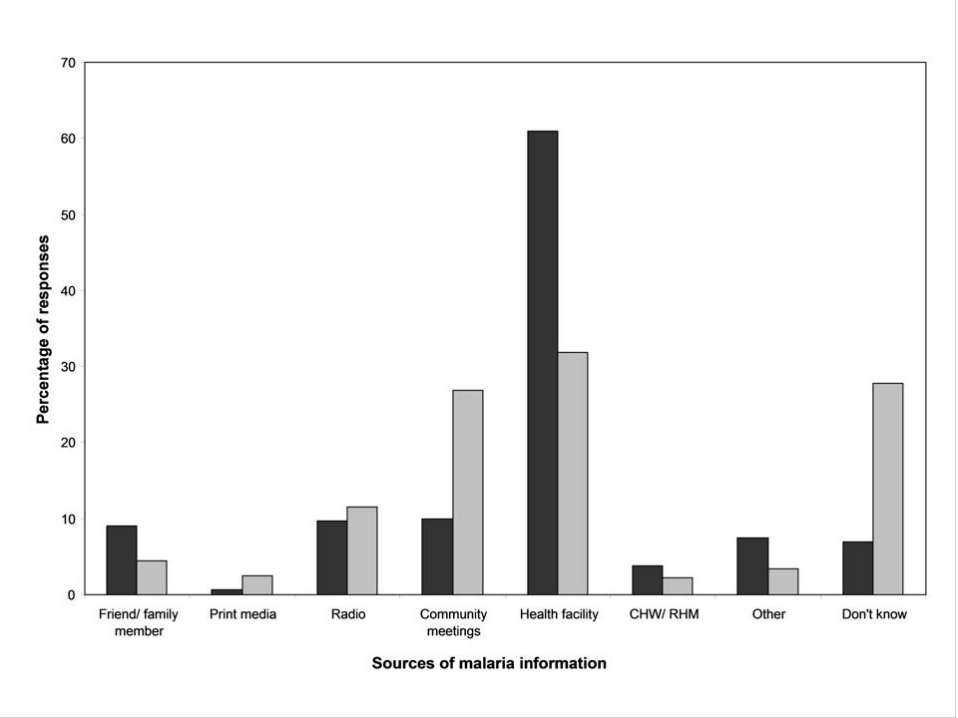
**Sources of malaria information (black bars) versus the preferred sources (grey bars) in the Lubombo region in Swaziland, CHWs denotes Community Health Workers and RHMs for Rural Health Motivators**.

Of 320 households surveyed 298 (93.1%) of the respondents had heard about malaria with almost all (99.7%, n = 297) of them correctly associating malaria with mosquito bites. All research participants that had heard about malaria demonstrated appropriate knowledge of and attitudes towards malaria by stating that it could kill if it is not treated. The participants gave a wide range of sources of malaria information as well as their preferences. Health facilities were the most prominent sources and community meetings were the second most preferred sources of information. However, very little malaria information came from community meetings. Likewise very little information came from the Community Health Workers (CHWs)/Rural Health Motivators (RHMs) (Figure 2).

**Figure 3:**
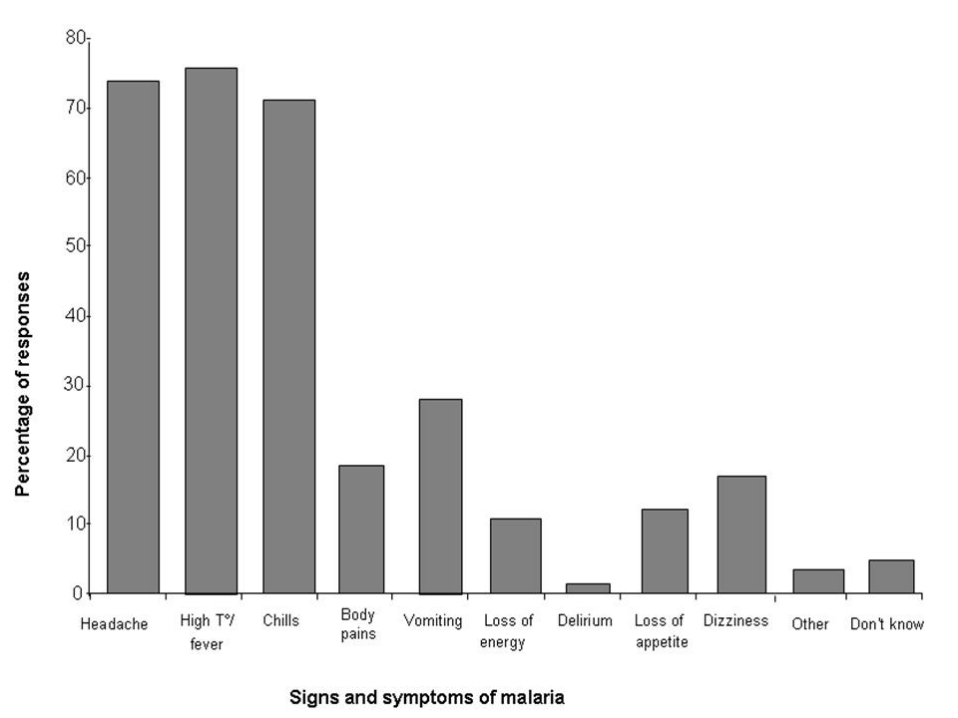
**Respondents' knowledge about signs and symptoms of malaria**.

Symptoms such as headache, high temperature/fever and chills were the three most frequently mentioned signs and symptoms of malaria. Although participants also identified loss of appetite and energy, dizziness and body pains the numbers were not convincing. A small proportion of the respondents included diarrhoea and cramps as other signs and symptoms of malaria (Figure 3).

### Malaria treatment-seeking behaviour and prevention

Knowledge about malaria treatment was high with 98.1% (n = 320) of the respondents stating that they would seek treatment in health facilities and 1.6% mentioned Rural Health Motivators (RHM). Only one research participant, who was also the head of household, presented no treatment-seeking plan. This respondent was 35 year old unemployed female and had no formal education.

Regarding households' promptness in seeking malaria treatment 88.1% (n = 320) stated that they would seek treatment within 24 hours of onset of malaria symptoms, with 8.8% reporting a delay of two to three days before seeking treatment. The remaining 3.1% presented no prompt treatment-seeking plan. The 8.8% of the respondents who stated that they would wait for 2 to 3 days were further asked about what they would do in the meantime while waiting to seek treatment. Half of them (14) stated that they would do nothing, while 28.6% (n = 28) would get painkillers, 10.7% (n = 28) would seek help from RHM and 10.7% (n = 28) would use oral rehydration with salt solution.

Generally, knowledge about malaria prevention among the participants was high 78% (n = 320), and only a small proportion (14.7%) said malaria can not be prevented and the remaining 7.3% of the participants did not know whether malaria is preventable. Most respondents knew that clinics and vector control are important for treating and preventing malaria disease. Another proportion mentioned hygiene, avoiding stagnant water in the yard, proper disposal of tins, continuous education and a small proportion thought that closing windows could help prevent malaria (Table 3). With regard to personal protective measures some participants stated that they use bed nets followed by mosquito coils and to a less extent the burning of cow dung/leaves, repellents sprays and lotion, but a substantial number of them did not use anything (Table 4).

**Table 3:** Respondents' knowledge and practices about malaria preventive measures in the Lubombo region, Swaziland, mid July 2007.

***Preventive measures***	***n***	***%***
Avoid stagnant water in the yard	11	4.4
Clinic	54	21.6
Close windows	3	1.2
Hygiene	31	12.4
Proper disposal of tins	15	6.0
Spraying	54	21.6
Through continuous education	11	4.4
Use bed nets	39	15.6
Other	6	2.4
Don't know	26	10.4

Total	250	100

**Table 4:** Respondents' knowledge and practices about personal protective measures against malaria in the Lubombo region, Swaziland, mid July 2007.

***Protective activities***	***n***	***%***
Use repellent lotion	15	4.7
Use mosquito coils	42	13.1
Use repellents sprays	12	3.8
Burn cow dung/leaves	11	3.4
Close windows & doors	20	6.3
Use mosquito nets	74	23.1
Other	27	8.4
Do nothing	139	43.4

### Perceptions about operational vector control activities

Out of the 320 surveyed households 87.2% of the respondents confirmed that spraying did take place during the 2006 transmission season. Of the 279 sprayed households 10% of the respondents had already replastered or painted the inner house walls by mid July 2007. Among the sprayed households, 91.4% (n = 279) of the respondents reported that the houses/rooms they sleep in were also sprayed as opposed to 6.1% not sprayed, and 2.5% did not know whether houses/rooms they sleep in were sprayed because they did not monitor the spraying activities. With regards to unsprayed households (12.5%) reasons were to prevent inconvenience, no spraymen came and occupants not available at the time of spraying. No explanations were provided in 11 unsprayed households. Information about the spraying season of 2006 could not be obtained only in one household.

Overall 38.8% (n = 124) of study households reported ownership of one or more bed nets (not checked whether treated or untreated). Among these, 46.8% were reported as belonging to children less than five years of age, 8.9% to children above five years of age, 43.5% to mothers and 4.8% to fathers. The recorded usage was 65.3%, and among the 34.8% of households, who did not use bed nets the reasons given were low mosquito population density and low disease incidence.

## Discussion

The potential contribution of KAP studies to malaria research and control has not received much attention in most Southern African countries. In Swaziland, this is the first study that has been carried out to provide baseline data about malaria related knowledge, attitude and practices at community level prior to the implementation of the malaria elimination strategy.

The results showed that most people had information about malaria. The most important source of information is health facilities. There was little information coming from the preferred source such as the *tinkhundlas *(traditional community district meetings). Similarly, measures promoted by the Department of Health such as community health workers (CHWs) and rural health motivators (RHMs) generated very little information about malaria. These are interesting results given the fact that malaria in this region is no longer endemic and compares well with studies in endemic countries such as Nigeria [7]. Hearing about malaria is not enough, but should be seen as a foundation through which a whole range of issues about malaria should be understood, for example, malaria transmission, signs and symptoms, prevention and treatment.

In this study, almost all (99.7%) of those who had heard about malaria made correct association between malaria and mosquito bite. These are encouraging results when compared to only 34% of people who made correct association in Zanzibar [8]. Investigations on communities' knowledge of signs and symptoms showed that over 70% of the respondents identified headache, high temperature/fever and chills as the most common ones. This is in line with the observations of most studies in endemic settings [9-11].

The analysis also showed that most respondents seek treatment in the health facilities. Contrary to most sub-Saharan African countries, where treatment is sought mainly in non-public sources [9,11-13], this difference could probably be attributed to better quality and accessibility of heath facilities in Swaziland compared to the other countries in the continent. Another interesting finding was that the majority (88.1%) of respondents in this study stated that they would seek treatment within 24 hours of onset of malaria symptoms. This exceeds the target defined by the Abuja summit on malaria, which says, *at least 60% of those suffering from malaria should seek treatment within 24 hours of the onset of symptoms' [14]. There was only one case where the respondent had no knowledge, and this was a household head with no education and employment.

Observations regarding preventive measures showed that most respondents (78.1%) believed that malaria is preventable, and mentioned, clinic, spraying and the use of bed nets as key malaria preventive measures. Despite these positive responses a substantial number of them (43.4%) did not take any personal protective measures to guard against malaria infection. This may be due to the fact that most people are dependant on interventions by the Malaria Control Programmes for protection against malaria infection.

The study found that 87.2% of the households had been sprayed during 2006 malaria season. This is in agreement with WHO guidelines on IRS coverage which recommends that it should be more than 80% within the targeted communities [4]. Only a small proportion of respondents were not happy about IRS. Interestingly there was also relatively good bed net coverage (38.8%), especially given the fact that IRS is the mainstay of malaria vector control in Swaziland. A small number of respondents stated that they did not use bed nets because of low mosquito population density and low disease incidence. Given the changing malaria situation in the country continued efforts are needed to emphasise the benefits of operational vector control activities for eliminating localized residual foci of transmission through community health promotion.

## Conclusion

In conclusion, most respondents showed an understanding of malaria transmission and its devastating effects. However, there is a need for improving the availability of information through the preferred community channels such as *tinkhundlas *as well as professional health routes such CHWs and RHMs. Furthermore, given the relatively moderate ownership of bed net there is a need for future studies to evaluate the distribution of insecticide treated nets compared with IRS. Although knowledge, attitudes and practices related to malaria in the study area are reasonable it has been demonstrated that as the level transmission and disease decreases so does the perception about the importance of malaria control activities [15]. The malaria elimination strategy should identify key socio-cultural and socio-economic indictors for monitoring progress. It is therefore recommended that other SADC countries earmarked for malaria elimination provide baseline information about local communities' perceptions and practices regarding malaria.

## Competing interests

The authors declare that they have no competing interests.

## Authors' contributions

KWH was involved in all study processes including design, data acquisition, analysis and interpretation of the results as well as the drafting of the manuscript. MLHM and RM contributed in the data analysis, initial and revised drafts of the manuscript. SK participated in the study design, data collection, the analysis and interpretation of the results. DG coordinated data capturing, data cleaning and data analysis.

## Supplementary Material

Additional file 1 **Additional file 1.** Household survey: KAP questionnaire (in English). Click here for file
