# Supplementary material for: Community knowledge, attitudes and practices (KAP) on malaria in Swaziland: A country earmarked for malaria elimination
Source: Malar J. 2009 Feb 19;8:29. doi: 10.1186/1475-2875-8-29 (PMC2649151; doi:10.1186/1475-2875-8-29)
Supplement: Additional file 1 — Household survey: KAP questionnaire (in English). [file 1475-2875-8-29-S1.pdf]

HOUSEHOLD SURVEY
KAP BASELINE 2007
SWAZILAND

NUMBER OF ENQUIRY

**Name of Interviewer**			*(signature)* ______________
**Date of interview**	/ / 2007		
**Starting (time)**			
**Ending (time)**			
**Interview time**			
**Verified by supervisor**	/ / 2007	h	*(signature)* ______________
**Code verified**	/ / 2007	h	*(signature)* ______________

**House number**	
**HH Head name**	
**No. of HH members**	
**Locality**	
**Inkundla**	
**Region**	

GPS points

S	
E	

INTRODUCTION

INDIVIDUAL CONSENT FOR THE INTERVIEW: DOMICILUM INVESTIGATION

Introduction: My name is …..I am conducting a research for the Department of Health about KAP in relation to malaria in this area. We would like to know various things about what you know about malaria issues. We are interviewing many people in this area.

Purpose of the research: The present research will help the malaria control program to better understand informational needs of the community on malaria issues.

Procedure: If you agree with the purpose of the research I will question you about your knowledge in relation to malaria . The questioning will last approx 15 minutes

Benefits: There are no direct benefits for you being part of this research. However your contributions will help the Malaria Control Programme and the Department of Health to design and develop appropraite information resources to help communities effectively recognise signs and symptoms of malaria and to take appropriate action when suspecting infection. You are free not to participate in this research or not to answer any question you feel uncomfortable with or even to abandon the questionnaire without consequences. Non-participation in the research does not imply that you will receive a different treatment at the healthcare facilities. Should you agree to be part of the research please feel free to interrupt the interview process at any time. Confidentiality is guaranteed and your answer will be part of many other household interviews so that your anonymity is ensured. We will not inform anyone of your participation in the research. Your name will not appear in any oral or written report of this study. There are no wrong or right answers. Your openness and honest opinions are extremely important. In case you do not understand a question or issue, please ask me to repeat or clarify.

1 **Would you like to participate in this interview?** [yes | no]
Relationship to the household head : ____

** In case the answer is* ***NO*** *thank them and* ***DO NOT*** *interview (do not mark, tick or circle anything in the questionnaire)*

HOUSEHOLD QUESTIONNAIRE

SECTION 1: HOUSEHOLD CENSUS

ID no. (Identification number for each family memb	2<br>Name<br>(Only first name for each family member)	3<br>Age<br>00= Don't know<br>In years	 <br><br>In months	4<br>Sex<br>1 = Male<br>2 = Female	5<br>Relationship to the HH head<br>1 = HH head<br>2 = Spouse<br>3 = Daughter/Son<br>4 = Daughter/ Son-in-law<br>5 = grandchild<br>6 = Parent<br>7 = Parent-in-law<br>8 =Grandparent<br>9 = Brother / sister<br>10 = Brother/ Sister-in-law<br>11 = friend<br>12 = lodger<br>13 = Other (specify)<br>98 = Not known	6<br>Highest level of education completed<br>1 = No education<br>2 = Creche - Grade 2<br>3 = Grade 3 - 7<br>4 = Grade 8 - 12<br>5 = Tertiary Student<br>6 = Tertiary Qualification<br>7 = Vocational School<br>8 = Night school<br>9 = Sebenta<br>10 = Other (specify)<br>11= Minor<br>98 = Don't know	7<br>Occupation of each person<br>1= Minor<br>2 = Unemployed<br>3 = Subsistence farming<br>4 = Farm worker<br>5 = Trained employee<br>6 = Small trader<br>7 = Civil servant<br>8 = Military<br>9 = Student<br>10 = Housewife<br>11 = Pensioner<br>12 = Other (specify)<br>98 = Don't know<br>99 = not applicable	8<br>Have you or any member of this household suffered from malaria this year (2007)?<br>1= Yes<br>2= No<br>98= Don't know
-1								
-2								
-3								
-4								
-5								
-6								
-7								
-8								
-9								
-10								
-11								
-12								

9. What type of dwellings/ structures are there in your household?

Type of Walls			Type of Roof		
1	Cane		1	Grass	
2	Canvas		2	Tiles	
3	Cement blocks		3	Asbestos	
4	Clay or clay blocks		4	Zinc	
5	Fire bricks		5	Canvas	
6	Stones and cement		6	Other (specify)	
7	Stones and mud				
8	Stick and mud				
9	Other (specify)				

INDIVIDUAL QUESTIONNAIRE

SECTION 2: MALARIA INFORMATION AND IEC

10. Have you heard about malaria?

- 1 Yes
- 2 No

11. If yes to question 10, where did you hear about malaria?

- 1 Friend
- 2 Family member
- 3 Posters/ pamphlets
- 4 Newspapers
- 5 Radio
- 6 TV
- 7 School
- 8 Church
- 9 Community meetings
- 10 Health facility
- 11 Community Health Workers/ RHM
- 12 Malaria Camp
- 13 Other (specify) ____________________
- 99 Not applicable

12. What transmits malaria? ....

13. Do you think malaria can kill you, if it is untreated?

- 1 Yes
- 2 No
- 98 Don't know
- 99 Not applicable

14. What do you think are the most common signs and symptoms in malaria infection?

- 1 Headache
- 2 High temperature/ fever
- 3 Body pains
- 4 Chills
- 5 Vomiting
- 6 Loss of energy
- 7 Delirium
- 8 Loss of appetite
- 9 Dizziness
- 10 Other (specify):...
- 98 Don't know
- 99 Not applicable

15. Do you think you have enough information on malaria?

- 1 Yes
- 2 No
- 98 Don't know

16. If no to question 15, what information would you like to get about malaria?

- 1 Information on treatment
- 2 Information on control
- 3 Information on prevention
- 4 Nature of the disease
- 5 Any information
- 6 Other (specify): ____________________
- 7 Signs and symptoms
- 98 Don't know
- 99 Not applicable

17. Where would you like this information communicated to you? *(Through what channels of communication?)*

- 1 Family member
- 2 Friend
- 3 Church
- 4 Radio
- 5 TV
- 6 Posters/ pamphlets
- 7 Newspapers
- 8 Health facility
- 9 Traditional healer
- 10 Community meetings
- 11 Community Health Workers/ RHM
- 12 Other (specify):..
- 98 Don't know
- 99 Not applicable

SECTION 3: TREATMENT AND TREATMENT-SEEKING BEHAVIOUR

18. If you or a member of your family were to present with the signs and symptoms of malaria where would you seek treatment?

- 1 Health facility
- 2 Traditional Healer
- 3 Pharmacy
- 4 No where
- 5 Other (specify):...
- 98 Don't know

19. How soon after suspecting that you are infected with malaria, would you seek treatment?

- 1 One day (within 24 hours)
- 2 2-3 days
- 3 4-6 days
- 4 7 days or more
- 99 Not applicable

20. If you would not seek treatment immediately (within 24 hours), what would you do? .... 99 Not applicable

SECTION 4: PERSONAL PROTECTION

21. Do you think malaria can be prevented? 1 Yes 2 No 98 Don't know

22. If yes to question 21, how? .... 99 Not applicable

23. What personal protective measures do you use to guard against malaria infection?

- 1 Use repellents
- 2 Use mosquito coils
- 3 Use doom
- 4 Burn cow dung/ leaves
- 5 Close windows & doors
- 6 Gauze wires in windows
- 7 Use mosquito nets
- 8 Other (specify):………………………………………….
- 9 Do nothing
- 99 Not applicable

24. Does this household have bednets? 1 Yes 4 No

25. If yes, who owns the available bednets in this household?

- 1 Father
- 2 Mother
- 3 Children over 5 years
- 4 Children under 5 years
- 6 Other *(specify)* :………………………………………….
- 99 Not applicable

26. Are all these bednets being used? 1 Yes 2 No 98 Don't know 99 Not applicable

27. If no to question 26, why? ……………………………………………………………… 98 Don't know 99 Not applicable

SECTION 5: SPRAYING AND MALARIA CONTROL PROGRAMME

28. Was your household sprayed last year (2006)? [1] Yes [2] No

29. If your household was not sprayed last year (2006), why?

1	Inconvenience	3	Spraymen not given permission to spray	98	Don't know	5	Other (specify): ____________
2	No one came to spray	4	No one at home	99	Not applicable		

30. If your household was sprayed last year (2006), was the house/ room you sleep in sprayed? [1] Yes [2] No [99] Not applicable

31. If your household was sprayed last year (2006), did sprayman explain the reasons for spraying? [1] Yes [2] No [99] Not applicable

32. Are you happy with the spraying service? [1] Yes [2] No [98] Don't know

33. If no to question 32, please give reasons?

1	Smell unsightly	4	Absence of household head	7	Spraymen's conduct	99	Not applicable
2	Inconvenience	5	Excites other insects (biting)	8	Discolouring house walls		
3	No malaria (or few cases)	6	Damage to belongings	9	Other (specify): ____________		

34. Were your inner house walls replastered or painted after the last year's (2006) spraying? [1] Yes [2] No [3] Forgot [99] Not applicable

We have concluded our interview. Thank you very much for your hospitality and your valuable contribution. Do you have any question for me?

____

____

OBSERVATIONS AND COMMENTS ABOUT THE INTERVIEW

35 Kindly describe the mood of the respondent(s) during the interview (indicate the time ended)

____

____

36 Did the other ones present during the interview participate in it? [yes] [no]

37 Any other observations? Any comment from debriefing?

____

____

____
